# Developmental high-risk criteria for severe mental illness: a neurodevelopmental framework for premorbid detection

**DOI:** 10.3389/fpsyt.2026.1829912

**Published:** 2026-06-23

**Authors:** Michele Poletti, Antonio Preti, Andrea Raballo

**Affiliations:** 1 Department of Mental Health and Pathological Addiction, Child and Adolescent Neuropsychiatry Service, Azienda USL-IRCCS di Reggio Emilia, Reggio Emilia, Italy; 2 Department of Neuroscience, University of Turin, Turin, Italy; 3 Chair of Psychiatry, Faculty of Biomedical Sciences, University of Southern Switzerland, Lugano, Switzerland; 4 Cantonal Sociopsychiatric Organisation, Mendrisio, Switzerland

**Keywords:** CAMHS, early detection, neurodevelopment, premorbid stage, prevention, prognosis, risk factors, severe mental illness

## Introduction

Severe mental illnesses (SMIs) such as schizophrenia, bipolar disorder, and major depressive disorder are increasingly conceptualized as neurodevelopmental in origin, i.e., clinical symptoms emerge during prodromal stage leaning on a neurobiological substrate of vulnerability unfolding along neurodevelopment, usually expressed in the phenotype in terms of unspecific premorbid features at the neurocognitive and behavioral levels (1). According to this perspective, rather than abruptly emerging during late adolescence or early adulthood, SMIs often evolve gradually through earlier, often subtle, developmental alterations reflecting complex dynamic interactions between individual and familial strengths and risk factors (2).

Contemporary models of psychiatric staging (3) suggest that the visible prodromal onset of attenuated psychotic or affective symptoms represents only the distal phase of a longer trajectory, which sometimes may be altered from birth or even during gestation (2). Moreover, only a fraction of help-seeking individuals intercepted by early detection services inspired by clinical high-risk models [for psychosis (CHR-P) or bipolar disorder (BAR)] undergo a homotypic transition to SMIs (i.e., from CHR-P to frank psychosis as well as from BAR to bipolar disorder) (4, 5); only a minority of adult patients with SMIs likewise were previously intercepted by CHR criteria (6).

To reach a wider audience of individuals who could potentially benefit from early prevention interventions, the focus of detection should shift from a narrow emphasis on attenuated or intermittent symptomatic prodromes to a broader, developmentally grounded profile of early (apparently unspecific) vulnerabilities (2, 7). There is accumulating evidence that the premorbid stage of several SMIs, usually coinciding with childhood and early adolescence, is concretely characterized by phenotypic manifestations that are unspecific as regards the prognostic outcome but signal the underlying atypical neurodevelopment—for example, childhood motor clumsiness, dyscoordination, and neurological soft signs have been associated with an increased likelihood of later psychotic outcomes at the group level in offspring of schizophrenic or bipolar parents (8), without implying deterministic or specific trajectories, and a childhood diagnosis of attention deficit/hyperactivity disorder is a precursor of later SMIs (9). Finally, the prognostic long-term salience of early neurodevelopmental manifestations for later SMIs is supported by register-based studies, showing a substantial proportion of future psychotic and bipolar disorder cases emerging in individuals who had attended child and adolescent mental health services (CAMHS) (10).

The prognostic salience of early neurodevelopmental phenotypic expressions was already caught, for example, by the Early Symptomatic Syndromes Eliciting Neurodevelopmental Clinical Examinations (ESSENCE) construct (11), profiled for children (and their parents) presenting in clinical settings with impairing child symptoms before age 3 (–5) years in the fields of (a) general development, (b) communication and language, (c) social inter-relatedness, (d) motor coordination, (e) attention, (f) activity, (g) behavior, (h) mood, and/or (i) sleep. However, with ESSENCE being a construct profiled for infancy, long-term prognostic outcomes up to adolescence or early adulthood are lacking.

Overall, early neurodevelopmental manifestations (due to the aggregation and interaction of genetic and early environmental risk factors) and later (if not concomitant) psychiatric manifestations could longitudinally represent two sides of the same coin along clinical staging (3), with the *p* factor (i.e., liability for psychopathology) putatively intercepting such vulnerability (12).

With the aim to operationally implement such shift from prodromal symptomatic manifestations to premorbid signals of risk, we attempt to profile a set of criteria for the construct of Developmental High-Risk for Severe Mental Illness (DHR-SMI) based on empirically supported and easily collectable features, not limited to neurodevelopmental expressivity but including also other risk factors that may cause or maintain such expressivity along time.

## DHR-SMI profile domains

The profile is based on five domains, shown in **Table 1** and operationalized in **Table 2**: three domains are based on risk factors that may affect neurodevelopment (presumed genetic risk, prenatal and perinatal hazards, and adverse childhood experiences), one domain is based on the severity of neurodevelopmental expressivity (CAMHS access and neurodevelopmental diagnoses), and one domain is based on sociodemographic vulnerability, which is capable of boosting stressing effects of the first three domains. Each domain can be scored 0/1/2 (absent/subthreshold/definite) to capture dose–response without forcing artificial cutoffs. The sum produces a 0–10 DHR-SMI score that can be treated as a continuous predictor in survival models. Such score can be profiled once as a baseline and longitudinally updated, considering that some domains are fixed (presumed genetic risk and prenatal/perinatal hazards) while others may undergo modifications along time.

**Table 1:** Developmental high-risk criteria for severe mental illness (DHR-SMI) profile.

DHR-SMI domain	Description
Presumed genetic risk	Familial psychiatric history: first- or second-degree parent with SMI or chronic psychopharmacological exposure
Prenatal and perinatal hazards	Prenatal infections or perinatal hazards (e.g., small for gestational age, low birth weight, caesarean delivery, hypoxia, low Apgar score) derived by medical records
Adverse childhood experiences (ACEs)	Verbal abuse, physical abuse, contact sexual abuse, a battered mother/father, household substance abuse, household mental illness, incarcerated household members, and parental separation or divorce. Both documented or self-reported on validated questionnaires on ACEs; ACEs can be split into early (0–5 years) vs. late (6–12 years) to test sensitive-period effects
CAMHS access	Access to child and adolescent mental health services and/or neurodevelopmental categorical diagnoses derived by medical records. CAMHS contacts can be weighted by frequency or cumulative service-days to avoid equating one visit with 5 years of care
Sociodemographic vulnerability	Immigrant status; urbanicity

Each domain can be scored 0/1/2 (absent/subthreshold/definite); the sum produces a 0–10 “DHR-SMI score”.

**Table 2:** Developmental high-risk criteria for severe mental illness (DHR-SMI) profile scoring grid.

Presumed genetic risk
Scores: 0 = none, 1 = 2nd-degree only, 2 = ≥1 1st-degree relative with SMI (SCZ, BD, MDD-psych, and schizoaffective) or chronic antipsychotic/mood stabilizer use >12 months
Prenatal/perinatal hazards
Extracted from the obstetric discharge summary: (i) birth weight <2,500 g, (ii) gestational age <37 weeks, (iii) 5-min Apgar <7, (iv) emergency C-section for fetal distress, (v) documented hypoxia/encephalopathy, and (vi) maternal infection requiring hospitalization during pregnancy
Scores: 0 = none, 1 = 1 hazard, 2 = ≥2 hazards
Adverse childhood experiences (ACEs)
10-item ACE questionnaire (parent-report for <10 years; self-report 10–12 years)
Each endorsed item = 1 point
Scores: 0 = none, 1 = 1–3 items, 2 = ≥4 items
CAMHS access and neurodevelopmental diagnosis
CAMHS referral or ICD-10/DSM-5 neurodevelopmental diagnosis (F70–F89 and F90–F98) already recorded
Scores: 0 = none, 1 = single contact or diagnosis (i.e., occasional contact), 2 = diagnosis and ≥3 contacts (persistent care management)
Additional sociodemographic risk factor
Immigration status = child or both parents born outside the host country
Urbanicity: living in a urban environment
Scores: 0 = not present, 1 = present

## Presumed genetic risk

Family psychiatric history remains one of the most reliable and accessible indicators of heritable vulnerability. Having a first-degree relative with SMI is associated with a significantly elevated risk of SMI in future offspring, i.e., in the most recent meta-analysis, The risk ratio and lifetime risk of developing any mental disorder were 3.0 and 55% in offspring of parents with anxiety disorders, 2.6 and 17% in offspring of those with psychosis, 2.1 and 55% in offspring of those with bipolar disorder, 1.9 and 51% in offspring of those with depressive disorders, and 1.5 and 38% in offspring of those with substance use disorders (13).

While polygenic risk scores currently show limited clinical utility due to modest predictive accuracy and the omission of complex transgenerational and epigenetic factors, family history encompasses both genetic and shared environmental contributions, rendering it a pragmatic marker in early risk stratification (14).

## Prenatal and perinatal hazards

Obstetric complications such as low birth weight, cesarean delivery, hypoxia, and prenatal infections represent early biological stressors that may disrupt normative brain development (15, 16). These insults can constrain neurodevelopment early in life, predisposing individuals to later childhood cognitive and emotional dysfunctions, which are core unspecific premorbid features of several SMIs (2, 7). Their routine documentation in perinatal medical records enhances their feasibility as screening variables in early risk assessment.

## Adverse childhood experiences

Early prolonged psychosocial stress, related to a dysfunctional familial environment and epidemiologically signaled by subjective reports of adverse childhood experiences in terms of abuse, maltreatment, and neglect, is consistently associated with elevated risk for psychotic and mood disorders (17). These adversities exert neurobiological effects through stress sensitization pathways and alterations in affect regulation, social cognition, and self-concept. Recognizing these experiences as foundational developmental disruptions rather than merely contextual variables is key to constructing comprehensive early risk profiles. Importantly, neurodevelopmental vulnerabilities may themselves increase exposure to adverse environments (e.g., through peer rejection, academic difficulties, or family stress), suggesting a bidirectional interaction between individual characteristics and environmental adversity across development. Along development, adverse experiences outside the familial environment, such as bullying or social rejection, also exacerbate subjective psychosocial stress and constraint intersubjectivity, contributing to the increased risk of mental illness (18). Conversely, early and targeted interventions (e.g., parenting support, school-based programs) may modify these trajectories, supporting functional reorganization and more adaptive developmental outcomes.

ACEs can be split into early (0–5 years) vs. late (6–12 years) to test sensitive-period effects.

## CAMHS access and neurodevelopmental diagnoses

Prior contact with CAMHS due to neurodevelopmental concerns—such as psychomotor delay, language impairment, learning disabilities, motor coordination difficulties, or behavioral disorders like ADHD —may reflect early, unspecific deviations in brain maturation. While often categorically diagnosed, these conditions may represent early manifestations of an underlying atypical brain development phenotypically expressed through unspecific manifestations of vulnerability (2, 7). Importantly, developmental psychopathology research highlights the heterotypic potential of early phenotypic patterns of neurodevelopmental vulnerability, i.e., multiple adolescent or adult mental health outcomes may derive from similar childhood clinical pictures.

Within this framework, early neurodevelopmental conditions (e.g., ADHD, language disorders, motor coordination difficulties) should not be interpreted as direct or deterministic precursors of later SMIs. Rather, they are conceptualized as potential expressions of a shared underlying neurodevelopmental vulnerability, which may evolve along multiple, heterogeneous trajectories. Lacking validated biomarkers of neurodevelopmental diagnoses, this may explain why many neurodevelopmental deviations compatible with categorical diagnoses of DSM-5 neurodevelopmental disorders (e.g. ADHD) are associated with higher prognostic and probabilistic risk of later multiple SMIs (8, 9).

Consistent with developmental psychopathology perspectives, these early manifestations are best understood in terms of probabilistic risk, shaped by interactions with environmental exposures, comorbidities, and access to timely interventions. This view also aligns with current nosographic frameworks, which distinguish neurodevelopmental disorders from severe mental illnesses while acknowledging their frequent co-occurrence and partial overlap in developmental pathways.

Thus, CAMHS data constitute a valuable resource for identifying at-risk children based on observable clinical features (10). CAMHS contacts can be weighted by length, frequency, or cumulative service-days to avoid equating one visit with 5 years of care.

## Sociodemographic vulnerability

Sociodemographic indicators such as immigrant status and urban residence have been independently linked to increased incidence rates of schizophrenia and other SMIs (19). These factors are hypothesized to interact with early neurobiological vulnerabilities via mechanisms involving social exclusion, discrimination, or environmental stress exposure. Given their accessibility through demographic data, they can be seamlessly integrated into early detection algorithms.

## Discussion

This integrative framework posits that early detection of risk for later SMI should emphasize the identification of early risk factors and of neurodevelopmental vulnerabilities during the putative childhood premorbid phase rather than rely solely on prodromal symptoms of later preclinical stage (2). The five proposed domains profiling DHR-SMI are not only accessible and operationalizable within existing mental health service infrastructures but also reflect empirically supported pathways of risk.

For example, a 10-year-old child presenting with a family history of mood disorder (score 2), low birth weight (score 1), exposure to early adversity (score 2), and repeated CAMHS contact for attentional and behavioral difficulties (score 2) would yield a DHR-SMI score of 7/10. In such a case, the framework would support enhanced monitoring and the implementation of preventive interventions targeting modifiable environmental factors rather than implying a deterministic clinical outcome.

The presence of one domain of risk may dimensionally increase the likelihood that other domains could be involved, considering the intertwining of many of them—for example, a familial psychiatric history involving a mother with SMI may increase both the likelihood of adverse childhood experiences due to maternal difficulties in being attuned to the relational needs of the infant and the likelihood of obstetric complications due to an inadequate lifestyle during pregnancy.

The early interaction of these three domains facilitates the emergence of phenotypic manifestations indexing a neurodevelopmental vulnerability and deserving CAMHS access. Considering multiple features profiling DHR-SMI, it is possible to hypothesize a kind of graded dose–response in terms of an association between the number of positive developmental features and the risk of subsequent SMI. From a dimensional perspective, the greater the probability of co-occurring features of the DHR-SMI profile, the less likely it is that pubertal development will alleviate or compensate these symptoms (prognostic persistence) (20). Such a neurodevelopmental profile of risk, profiling the early aggregation of genetic and environmental risk factors as well as their early phenotypic expressivity, could be central to the early premorbid detection of risk for later psychiatric manifestations and to early preventive intervention aimed at mitigating prognostic risk by focusing on modifiable risk factors such as parenting. Keeping in mind the heterotypic potential of many childhood neurodevelopmental conditions is crucial to prognostically conceptualize their possible trajectories toward adult SMI through adolescent prodromes.

Importantly, the DHR-SMI framework is not intended as a static snapshot of risk but as a dynamic tool that can be iteratively updated across development. While some domains (e.g., genetic risk, prenatal/perinatal hazards) remain stable, others (e.g., adverse experiences, CAMHS engagement, sociodemographic conditions) may change over time, reflecting ongoing interactions between the individual and the environment. A longitudinal reassessment of the DHR-SMI profile may therefore capture evolving developmental trajectories, including both risk amplification and risk attenuation in response to environmental changes or clinical interventions. In this perspective, the model may complement developmental staging approaches (3) by providing a scalable and updateable index of cumulative vulnerability across time.

Lastly, this risk profiling has also limitations, including possible biases inherent in the scoring of each risk domain, as well as the poor consideration of protective factors, hampering the possible testing of moderating factors. As regards scoring, the additive structure of the DHR-SMI score is primarily intended as a pragmatic and clinically feasible approach rather than a fully empirically calibrated weighting system. At the current stage, domains are equally weighted to ensure ease of use and transparency in routine settings. However, we acknowledge that different risk factors may carry differential predictive value. Future empirical studies are needed to refine the weighting of domains and thresholds based on longitudinal predictive performance, potentially leading to data-driven recalibration of the scoring system. It is also important to note that single high-impact factors (e.g., very low birth weight or severe perinatal complications) may carry substantial individual risk, even when not accompanied by additional factors. The current scoring system prioritizes cumulative burden but does not preclude the clinical relevance of specific high-risk exposures.

Overall, it is important to clarify that the high sensitivity of the proposed framework refers to the identification, among help-seeking individuals, of those showing enhanced developmental risk. Such risk has pleiotropic implications for later biopsychosocial functioning and quality of life, even if it does not converge on a single categorical diagnosis. Thus, while specificity is indeed limited with respect to predicting discrete diagnostic outcomes, it is substantial with respect to forecasting broader trajectories of suboptimal mental health and functional impairment. From this perspective, the value of developmental profiling lies less in categorical prognostication and more in stratifying individuals at risk for enduring clinical needs. Future technological advances—such as polygenic risk modeling, neuroimaging, and electrophysiological biomarkers, digital phenotyping, and AI-driven integration of longitudinal datasets—may further enhance specificity, particularly for diagnostic differentiation, thereby complementing the developmental high-risk framework.

In summary, this prognostic model is inherently characterized by high sensitivity and low specificity, as it targets the prospective risk of suboptimal outcomes and poor quality of life. It is therefore designed to support early, broadly defined psychosocial and developmental interventions in help-seeking contexts, coupled with ongoing monitoring to prevent further deterioration. Thus, the aim is not to avert a specific, homotypic outcome—as in the clinical high-risk state for psychosis—but to address a broader, pleiotropic risk across the lifespan.

## Conclusions

A developmentally sensitive and transdiagnostic profiling model could facilitate the early identification of individuals on high-risk trajectories, thereby supporting tailored monitoring and targeted interventions during critical windows of plasticity. Expanding the focus of psychiatric risk prediction to encompass premorbid developmental markers offers a promising avenue for improving the timing and effectiveness of preventive interventions. The proposed five-domain framework—spanning genetic, biological, psychosocial, demographic, and clinical service indicators—enables a multidimensional and feasible approach to early detection. Such a model, which needs field testing as regards feasibility and predictive validity, may ultimately enhance the precision of psychiatric prognostication and inform the design of stage-specific intervention strategies.
